# Interkulturelle Adaptation der PUKSoPC in deutscher Sprache: Eine Skala zur gefühlten Kontrolle von Parkinson-Patientinnen und -Patienten

**DOI:** 10.1007/s00115-023-01569-2

**Published:** 2023-11-20

**Authors:** V. Stopic, A. Rizos, J. Simpson, F. J. R. Eccles, T. A. Dembek, M. T. Barbe, A. Sauerbier

**Affiliations:** 1 Klinik und Poliklinik für Neurologie, Universitätsklinik Köln, Kerpener Str. 62, 50937 Köln, Deutschland; 2 Department of Neurology, King’s College Hospital NHS Foundation Trust, London, Großbritannien; 3 Division of Health Research, Faculty of Health and Medicine, Lancaster University, Lancaster, Großbritannien; 4 Institute of Psychiatry, Psychology and Neuroscience, King’s College London, London, Großbritannien

**Keywords:** Parkinson-Erkrankung, Gefühlte Kontrolle, Skala, Übersetzung, Validierung, Parkinson’s disease, Perceived control, Scale, Translation, Validation

## Abstract

**Hintergrund:**

Die gefühlte Kontrolle von Menschen mit Parkinson-Erkrankung spielt eine große Rolle für ihre Lebensqualität. Simpson et al. entwickelten eine für die Parkinson-Erkrankung spezifische Skala der gefühlten Kontrolle namens Parkinson’s UK Scale of Perceived Control (PUKSoPC). Wir stellen in dieser Arbeit eine interkulturell adaptierte deutsche Übersetzung der englischen Originalversion vor.

**Methoden:**

Nach Zustimmung der Originalautoren wurde ein international etabliertes Prozedere für die interkulturelle Adaptation eingesetzt. Die englischsprachige Originalversion wurde unabhängig von zwei bilingualen Neurowissenschaftlern übersetzt und anschließend von beiden eine Konsensusversion gebildet. Diese wurde an 10 Parkinson-Patientinnen und -Patienten getestet und von zwei weiteren Neurowissenschaftlern unabhängig in die englische Sprache rückübersetzt. Nach Bildung einer Konsensusversion wurde diese englische Version von allen vier Übersetzern mit der Originalversion verglichen. Differenzen zwischen den Versionen resultierten in Modifikationen der deutschen Übersetzung, sodass die Rückübersetzung möglichst genau dem Original entsprach. Die finale Version wurde von zwei der Originalautoren genehmigt und an 50 Parkinson-Patientinnen und -Patienten klinisch getestet.

**Ergebnisse:**

Im Rahmen des Übersetzungsprozesses einigten sich die vier Übersetzer auf eine kulturell adaptierte deutsche Fassung der PUKSoPC. Bei der Testung der finalen Version an 50 Parkinson-Patientinnen und -Patienten zeigten sich keine sprachlichen oder inhaltlichen Probleme.

**Diskussion:**

Die vorgestellte, sprachlich validierte deutsche Version der PUKSoPC steht nun zur Erhebung der gefühlten Kontrolle von Parkinson-Patientinnen und -Patienten in Forschung und klinischem Alltag zur Verfügung.

## Abstract (en)

**Background:**

The level of perceived control in people with Parkinson’s disease plays a significant role in affecting their quality of life. Simpson et al. developed a scale of perceived control specific to Parkinson’s disease called the Parkinson’s UK Scale of Perceived Control (PUKSoPC). In this work, we present a cross-culturally adapted German translation of the original English version.

**Methods:**

After receiving approval by the original authors, an internationally established procedure was used for cross-cultural adaptation. Firstly, the original English version was translated into German independently by two bilingual neuroscientists, who then agreed on a consensus version. This was tested on 10 people with Parkinson’s disease and independently back translated into English by two different neuroscientists. After forming a consensus version, this English version was compared with the original version by all four translators. Differences between the versions resulted in modifications to the German translation so that the back translation matched the original as closely as possible. The final version was approved by two of the original authors and clinically tested on 50 people with Parkinson’s disease.

**Results:**

During the translation process, the four translators agreed on a culturally adapted German version of the PUKSoPC. Testing of the final version on 50 people with Parkinson’s disease did not reveal any linguistic or content-related problems.

**Conclusion:**

The linguistically validated German version of the PUKSoPC presented in this paper is now freely available for measuring the levels of perceived control in people with Parkinson’s disease to advance both research and clinical practice.

Gefühlte Kontrolle hat einen großen Einfluss auf das psychische Wohlbefinden von Menschen mit Parkinson-Erkrankung. Mit der Parkinson’s UK (United Kingdom) Skala der gefühlten Kontrolle (Parkinson’s UK Scale of Perceived Control, PUKSoPC) wurde die erste Skala entwickelt, welche die gefühlte Kontrolle von Parkinson-Patientinnen und -Patienten krankheitsspezifisch erfasst. Da die Skala bislang nur in englischer Sprache vorlag, haben wir diese gemäß international anerkannten Vorgaben übersetzt und interkulturell adaptiert. In dieser Arbeit wird der Übersetzungsprozess sowie die validierte deutsche Version vorgestellt.

Das Konzept der gefühlten Kontrolle spielt eine wichtige Rolle bei chronischen Erkrankungen wie der Parkinson-Erkrankung [12]. Sie wird definiert als „die Überzeugung, dass man seine eigenen internen Zustände und Verhalten bestimmen, seine Umgebung beeinflussen und/oder gewünschte Ergebnisse erzielen kann“ [12]. Die gefühlte Kontrolle stellt einen bedeutsamen Faktor bei der Adaptation an eine chronische Erkrankung dar [5, 10]. Ein hoher Grad an gefühlter Kontrolle bei Parkinson-Patientinnen und -Patienten ist assoziiert mit hoher Lebensqualität, der genauen Einhaltung der Medikamenteneinnahme sowie geringer Ausprägung einer Depression [13]. Trotz der hohen Spezifität der gefühlten Kontrolle bei der Parkinson-Erkrankung wurden in der Vergangenheit lediglich krankheitsübergreifende Instrumente zur Messung verwendet [2, 8]. Aus diesem Grund entwickelten Simpson et al. eine für die Parkinson-Erkrankung spezifische Skala der gefühlten Kontrolle namens Parkinson’s UK Scale of Perceived Control (PUKSoPC), welche bisher nur in der Originalversion auf Englisch vorlag [9]. Die selbstberichte Skala besteht aus 15 Aussagen in den folgenden 5 Teilskalen mit jeweils 3 Aussagen: „Positiv denken“, „Sich informieren“, „Dinge tun“, „Planen“ und „Engagiert sein“. Die Zustimmung zu den einzelnen Aussagen soll auf einer Skala von 1 (gar nicht) bis 5 (ganz genau) bewertet werden.

Wir stellen in dieser Arbeit eine gemäß international anerkannten Vorgaben erstellte, interkulturell adaptierte deutsche Übersetzung vor.

## Methoden

Vor Beginn der Übersetzung wurde das Einverständnis der Autoren der Originalversion des PUKSoPCs zum Vorhaben eingeholt (JS, FE). Zur Adaptation in deutscher Sprache wurde ein international anerkanntes Prozedere durchlaufen [1, 4]: Als erstes wurde die englischsprachige Originalversion unabhängig von zwei bilingualen Neurowissenschaftlern mit Expertise im Bereich der Parkinson-Erkrankung (AR, AS) möglichst wörtlich in die deutsche Sprache übersetzt. Anschließend wurden beide Versionen von den Übersetzern verglichen und eine Konsensusversion erstellt. Um mögliche erste Verständnisprobleme zu beseitigen, wurde diese Version bei 10 Patientinnen und Patienten mit idiopathischer Parkinson-Erkrankung (Diagnose gemäß UK-Brain-Bank-Kriterien) am Universitätsklinikum Köln klinisch getestet (VS). Die Kommentare der Patientinnen und Patienten wurden in die deutsche Fassung eingearbeitet. Letztere wurde anschließend von zwei weiteren bilingualen Neurowissenschaftlern mit Expertise im Bereich der Parkinson-Erkrankung (TD, MB) unabhängig voneinander in die englische Originalsprache zurückübersetzt. Nach Abgleich der Versionen wurde von den Übersetzern eine englische Konsensusversion erstellt. Diese wurde anschließend von allen beteiligten Übersetzern (AR, AS, TD, MB) mit der Originalversion verglichen. In diesem Rahmen wurden geringe Differenzen zwischen beiden englischen Versionen aufgedeckt. Bei abweichend übersetzten Wörtern in der englischen Rückübersetzung wurden die Wörter in der deutschen Version noch einmal betrachtet und durch Synonyme ersetzt, die den Sinn der Wörter in der Originalversion besser widerspiegelten. Die finale deutsche Übersetzung sowie die englische Rückübersetzung wurden zudem an zwei der Autoren der Originalarbeit (JS, FE) geschickt und von diesen genehmigt. Zuletzt wurde die finale Version an 50 Patientinnen und Patienten mit idiopathischer Parkinson-Erkrankung (Diagnose gemäß UK-Brain-Bank-Kriterien) am Universitätsklinikum Köln klinisch getestet (VS, AS), um mögliche sprachliche und inhaltliche Probleme zu erkennen.

## Ergebnisse

Die erste Testung der deutschen Version an 10 Patientinnen und Patienten mit idiopathischer Parkinson-Erkrankung (VS) ergab keine sprachlichen oder inhaltlichen Probleme. Lediglich Aussage 10 „Ich habe Mittel und Wege, die mir helfen, daran zu denken, was ich tun wollte“ wurde von einer Patientin aufgrund der Länge als etwas unleserlich erachtet. Leider ist es im Deutschen grammatikalisch nicht möglich, eine kürzere Version des Satzes zu finden, ohne den Sinn abzuändern. Daher wurde der Satz beibehalten. Bei der Findung einer Konsensusversion durch alle vier Übersetzer stellte sich heraus, dass einige Wörter in der englischen Sprache anders verwendet werden als in der deutschen Sprache. Die Übersetzung des Titels der Skala war zunächst nicht ganz eindeutig, da der Begriff „perceived control“ in diesem Kontext im Englischen eine affektive Komponente enthält, die in der Übersetzung „wahrgenommene Kontrolle“ nicht ganz deutlich wird. Aus diesem Grund wurde als Übersetzung „gefühlte Kontrolle“ gewählt. Die Antwortoptionen „quite a lot“ und „very much“ wurden mit „ziemlich genau“ und „ganz genau“ übersetzt. Angepasst an den deutschen Sprachraum wurde „Parkinson’s“ mit „Parkinson-Erkrankung“ übersetzt. Die wörtliche Übersetzung des Ausdrucks „remember to do things“ in Aussage 10 „sich erinnern, Dinge zu tun“ wird in der deutschen Sprache so nicht verwendet, sodass die Übersetzung „sich erinnern, was man tun wollte“ gewählt wurde. Als nationale Organisation, bei welcher man sich als Patientin oder Patient engagieren kann, wurde in der Originalversion in Aussage 15 als Beispiel „Parkinson’s UK“ gewählt, welche durch die in Deutschland bekannte „Deutsche Parkinson Vereinigung“ ersetzt wurde.

Die Zusendung der finalen deutschen Übersetzung sowie englischen Rückübersetzung an zwei der Autoren der Originalarbeit (JS, FE) resultierte in einigen wenigen Anmerkungen zur englischen Rückübersetzung, welche jedoch dadurch entkräftet wurden, dass die deutsche Version als sprachlich passend erachtet wurde.

**Figure 1:**
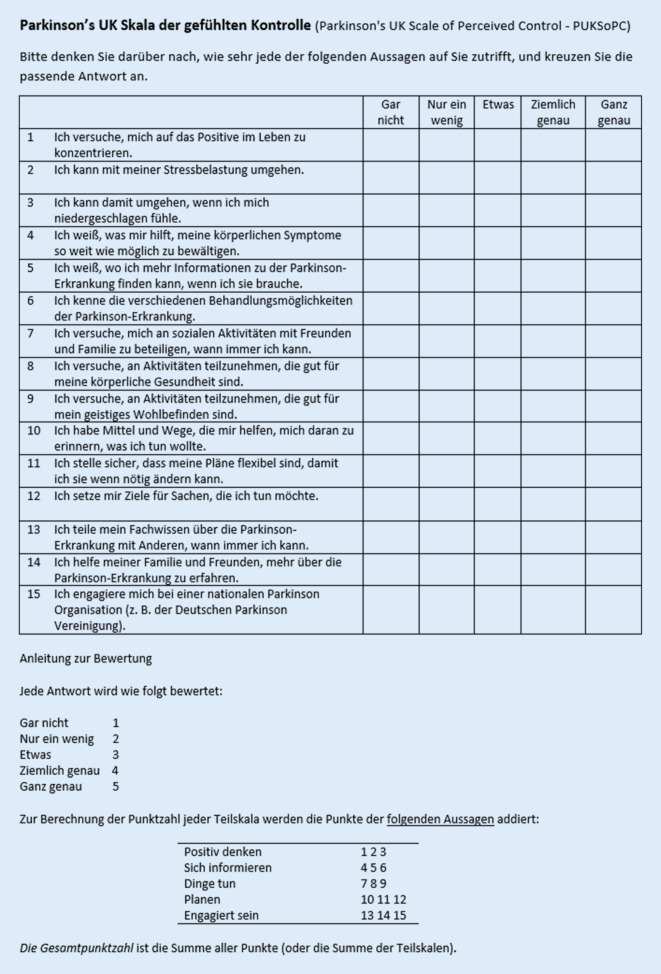

Bei der Testung der finalen Version der deutschen Übersetzung des PUKSoPCs an 50 Patientinnen und Patienten mit idiopathischer Parkinson-Erkrankung (VS, AS) zeigten sich keinerlei sprachliche oder inhaltliche Probleme. Die finale, interkulturell adaptierte deutsche Übersetzung ist in Abb. 1 gezeigt.

## Diskussion

In dieser Arbeit wurde eine interkulturell adaptierte deutsche Übersetzung der PUKSoPC, einer Skala zur gefühlten Kontrolle von Patientinnen und Patienten mit idiopathischer Parkinson-Erkrankung, vorgestellt. Diese wurde auf Basis international anerkannter Vorgaben für interkulturelle Übersetzungen von Fragebögen und Skalen erstellt [1, 4]. Die Testung der finalen Version zeigte keinerlei sprachliche oder inhaltliche Probleme. Daher kann angenommen werden, dass sie ebenfalls wie die validierte Originalversion eine gute Augenscheinvalidität, Test-Retest-Reliabilität sowie solide Faktorenstruktur aufweist.

Mit der Entwicklung der PUKSoPC wurde erstmals eine Skala vorgestellt, welche die gefühlte Kontrolle von Patientinnen und Patienten mit idiopathischer Parkinson-Erkrankung erfasst. Unter Nutzung der Skala bei dieser Patientengruppe konnte bereits gefunden werden, dass gefühlte Kontrolle als Mediator fungiert zwischen Stigma und Lebensqualität sowie Stigma und Depression [11]. Das Konstrukt des Stigmas spielt bei der Parkinson-Erkrankung ebenfalls eine wichtige Rolle: Es bezeichnet das gefühlte oder tatsächliche Auftreten von Labeling, Stereotypen, Ausgrenzung, Statusverlust oder Diskriminierung aufgrund der Parkinson-Erkrankung [3, 6, 7]. Somit kann ein hohes Maß an gefühlter Kontrolle den negativen Effekt, den ein hohes Maß an Stigma auf die Lebensqualität und Depression hat, abschwächen. Ebenfalls wurde gezeigt, dass gefühlte Kontrolle u. a. mit psychologischer Unterstützung, Wissen über Medikamente sowie Alter und Geschlecht assoziiert ist [13]. Auf Grundlage dieser Erkenntnisse könnten solche Einflussvariablen modifiziert werden, was zu einer Steigerung der gefühlten Kontrolle und damit wiederum zu einer Verbesserung der Lebensqualität der Patientinnen und Patienten mit idiopathischer Parkinson-Erkrankung führen kann.

Da bislang nur die englische Originalversion zur Nutzung in der Forschung zur Verfügung stand, beruhen diese Erkenntnisse lediglich auf Studien, welche im englischsprachigen Raum durchgeführt wurden. Da es naheliegend ist, dass das Konzept der gefühlten Kontrolle allgemein von großer Bedeutung bei der Parkinson-Erkrankung ist, sollte es auch möglich sein, die gefühlte Kontrolle in deutschsprachigen Regionen routinemäßig in klinischer Praxis und klinischen Studien erheben zu können. Aus diesem Grund stellen wir hiermit eine kulturell adaptierte, deutsche Übersetzung der PUKSoPC vor, welche kostenlos zur Verfügung steht. Eventuell offenbaren sich in deutschsprachigen Regionen aufgrund interkultureller Unterschiede andere Einflussfaktoren und Zusammenhänge als zuvor gefunden werden konnten. Die Erforschung möglicher Unterschiede in der Ausprägung der gefühlten Kontrolle wird ebenfalls durch unsere Übersetzung ermöglicht. Die Nutzung eigenständig übersetzter Versionen ist daher nicht mehr notwendig oder angebracht.

Die deutsche Übersetzung kann kostenlos auf Anfrage an den korrespondierenden Autor als PDF-Version bezogen werden.

## Fazit für die Praxis

- Die Parkinson’s UK Scale of Perceived Control (PUKSoPC) ist die erste krankheitsspezifische Skala zur Messung der gefühlten Kontrolle bei Patientinnen und Patienten mit Parkinson-Erkrankung.
- Wir stellen in dieser Arbeit eine kulturell adaptierte deutsche Übersetzung der im Original in englischer Sprache verfassten Skala vor, welche kostenlos für den Einsatz in klinischer Praxis und Forschung verfügbar ist.
- Durch Nutzung international anerkannter Vorgaben bei der interkulturellen Adaptation kann von vergleichbaren psychometrischen Eigenschaften der Übersetzung ausgegangen werden.
